# Expression of NF-κB associated lncRNAs in schizophrenia

**DOI:** 10.1038/s41598-020-75333-w

**Published:** 2020-10-22

**Authors:** Amin Safa, Elham Badrlou, Shahram Arsang-Jang, Arezou Sayad, Mohammad Taheri, Soudeh Ghafouri-Fard

**Affiliations:** 1 Institute of Research and Development, Duy Tan University, Da Nang, 550000 Vietnam; 2 Department of Immunology, School of Medicine, Zabol University of Medical Sciences, Zabol, Iran; 3 Department of Medical Genetics, Shahid Beheshti University of Medical Sciences, Tehran, Iran; 4 Department of Biostatistics and Epidemiology, Cancer Gene Therapy Research Center, Faculty of Medicine, Zanjan University of Medical Sciences, Zanjan, Iran; 5 Urogenital Stem Cell Research Center, Shahid Beheshti University of Medical Sciences, Tehran, Iran

**Keywords:** Epigenetics, Gene expression, Genetic markers

## Abstract

NF-κB signaling pathway has important roles in the regulation of growth and development of nervous system. This pathway has also been shown to participate in the pathogenesis of schizophrenia. Meanwhile, activity of NF-κB signaling pathway is regulated by several factors including non-coding RNAs (lncRNAs). In the current study, we evaluated expression of nine NF-κB-related lncRNAs namely *DILC*, *ANRIL*, *PACER*, *CHAST*, *ADINR*, *DICER1-AS1*, *HNF1A-AS1*, *H19* and *NKILA* as well as two mRNA coding genes namely *ATG5* and *CEBPA* in the peripheral blood of patients with schizophrenia compared with matched healthy subjects. Expressions of these genes were assessed by real time PCR technique. Expression of *PACER* was lower in patients with schizophrenia compared with controls (Posterior beta = − 0.684, P value = 0.049). On the other hand, expressions of *CHAST*, *CEBPA*, *H19*, *HNF1A-AS1* and *DICER1-AS1* were higher in patients compared with controls (Posterior beta = 0.39, P value = 0.005; Posterior beta = 0.844, P value < 0.0001; Posterior beta = 0.467, P value < 0.0001; Posterior beta = 1.107, P value = 0.005; Posterior beta = 0.176, P value = 0.044, respectively). We also appraised the diagnostic power of transcript quantities of *CHAST*, *CEBPA*, *DICER1-AS1*, *H19* and *HNF1A-AS1* in distinguishing between patients with schizophrenia and controls through depicting ROC curves. Based on the area under curve (AUC) values, *CEBPA* had the best diagnostic power (AUC = 0.948, P < 0.0001), followed by *H19* (AUC = 0.815, P < 0.0001). Taken together, our study demonstrated dysregulation of NF-κB-related lncRNAs and genes in the peripheral blood of patients with schizophrenia and their potential as peripheral markers for this psychiatric condition.

## Introduction

The nuclear factor-κB (NF-κB) family of proteins includes a number of proteins all of which having a conserved DNA-binding/dimerization domain namely the Rel homology domain (RHD)^1^. In addition to DNA binding and dimerization, RHD mediates binding with the inhibitor of NF-κB (IκB) protein and nuclear translocation of NF-κB^2^. NF-κB signaling pathway has crucial roles in the regulation of growth and development of nervous system^3^. This pathway modulates expression of genes participating in the inflammatory responses, cell survival and plasticity of neurons. In the course of neurodevelopment, NF-κB signaling facilitates the influences of several biomolecules including cytokines, chemokines, growth factors and extracellular matrix proteins in the neuronal niche^2^. NF-κB is a fundamental facilitator of pro-inflammatory gene activation and stimulation in both innate and adaptive immune cells^4^. Pro-inflammatory cytokines, chemokines and other inflammatory molecules induced by NF-κB pathway promote inflammation through both direct routes and indirect enhancement of differentiation of inflammatory T cells^4^. In addition, the interactions between NF-κB signaling pathway and Notch, Shh and Wnt/β-catenin pathways has crucial roles in the neurodevelopmental processes^2^. Consistent with the important role of this pathway in neuronal plasticity, a previous study has demonstrated down-regulation of NF-κB expression and decreased nuclear activation of this factor in the superior temporal gyrus of patients with schizophrenia^5^. Moreover, genes regulating the translocation of NF-κB have been dysregulated in these patients^5^. Activity of NF‐κB signaling pathway and sub-cellular localization of this factor are regulated by several mechanisms among them is the epigenetic modulation by long non‐coding RNAs (lncRNAs)^6^. Based on the previously reported dysregulation of NF-κB signaling in schizophrenia^5^ and the regulatory role of lncRNAs on this pathway^6^, in the current study, we evaluated expression of nine NF-κB-related lncRNAs namely *DILC*, *ANRIL*, *PACER*, *CHAST*, *ADINR*, *DICER1-AS1*, *HNF1A-AS1*, *H19* and *NKILA* as well as two mRNA coding genes namely *ATG5* and *CEBPA* in the peripheral blood of patients with schizophrenia compared with matched healthy subjects. The basis for selection of these lncRNAs and mRNA coding genes was their functional relevance with NF-κB signaling. Expression of some of these genes are regulated by NF-κB. For instance, expression of *NKILA* has been shown to be activated by NF-κB. This lncRNA binds with NF-κB/IκB, and conceals phosphorylation sites of IκB, therefore inhibiting NF-κB activation^7^. Others indirectly regulate the interaction between NF-κB and other signaling pathways or modulate the role of NF-κB in cellular functions. The lncRNA *DILC* participates in the regulation of interaction between TNF-α/NF-κB signaling and IL-6/STAT3 axis^8^. *ANRIL* has been identified as an important element in the NF-κB pathway that regulates inflammatory responses^9^. *PACER* has been shown to induce COX-2 gene expression through blocking suppressive NF-κB complexes^10^. *CHAST* is an lncRNA which influences the activity of Wnt signaling^11^, a pathway that is functionally linked with NF-κB signaling^12^. *ADINR* is an lncRNA transcribed from a genomic region near of the *CEBPA* gene and its expression is correlated with the expression its nearby gene^13^. CEBPA participates in the induction of NF-κB target genes through replacement of histone deacetylases from NF-κB p50 homodimers^14^. *DICER1-AS1* has been shown to regulate autophagy via modulation of miR-30b/ATG5 axis^15^. Notably, autophagy contributes in several cellular functions related with NF-κB signaling^16^. *HNF1A-AS1* is an lncRNA which is transcribed from the opposite direction of the *HNF1A* gene^17^, a transcription factor that interacts with the NF-κB signaling^18^. Finally, several studies have reported the interaction between *H19* and NF-κB signaling in diverse situations^19,20^. Based on the prominent roles of these genes in the regulation of NF-κB and involvement of this pathway in neurodevelopment and inflammation, we hypothesized that expression of these geens have been dysregulated in the peripheral blood of patients with schizophrenia as a reflection of their expression in the central tissues.

## Materials and methods

### Study participants

The present project was implemented in samples obtained from 50 patients with schizophrenia (33 male patients and 17 female patients, mean age ± standard deviation: 49.62 ± 9.63) and 50 healthy subjects (33 male patients and 17 female patients, mean age ± standard deviation: 49.78 ± 11.94). Cases were recruited from hospitals affiliated with Shahid Beheshti and Hamadan Universities of Medical Sciences. The fifth edition of Diagnostic and Statistical Manual of Mental Disorders (DSM-V) was applied in the diagnostic process^21,22^. The standard dose of Clozapine (301 mg/day to 600 mg/day) was used for treatment of patients. Patients were included in the study if their symptoms/signs were in conformity with the mentioned diagnostic criteria. Those with current substance abuse or cigarette smoking were excluded from the study. The Mini-International Neuropsychiatric Interview^23^ was used for assessment of control subjects. Exclusion criteria for this study group were the existence of systemic disorders, psychiatric conditions or pregnancy. Moreover, we have excluded healthy controls who had a first-degree biological relative with a history of psychopathology. The study protocol was approved by Ethical Committee of Shahid Beheshti University of Medical Sciences and all methods were performed in accordance with the relevant guidelines and regulations. Informed written consent forms were signed by all study participants.

### Expression assay

Five milliliters of the peripheral blood were gathered in EDTA-containing falcon tubes. These specimens were subjected to RNA extraction by the Hybrid-R blood RNA extraction Kit (GeneAll, Seoul, Korea). All steps were accomplished according to the manufacturer’s protocol. The yielded RNA was subsequently converted to cDNA by using the High-Capacity cDNA Reverse Transcription Kit (Thermo Fisher Scientific, Gent, Belgium). Expressions of mentioned lncRNAs were assessed in all enrolled individuals using the RealQ Plus 2 × PCR Master Mix Green Without ROX PCR Master Mix (Ampliqon, Odense, Denmark). Cycling reactions were carried out in Step One Plus Real-Time PCR equipment (Applied Biosystems, Foster city, CA, USA). Primers used for expression assays and PCR products sizes were identical to our previous studies^24,25^. Table S1 summarizes the primers features. We have chosen *B2M* gene as the reference gene based on our previous observations regrading constant expression of this gene in peripheral blood of patients with schizophrenia^26^.

### Statistical methods

Relative transcripts levels of lncRNAs were measured in all samples the Ln [Efficiency^ΔCT] method considering the transcript levels of B2M as normalizer. Hierarchical Bayesian regression model was used for comparison of these values between cases and controls. The impacts of independent variables were adjusted. The asymmetric Laplace prior distribution was supposed with 4000 iteration and 1000 warm-ups for parameterization of expression ratio of lncRNAs/genes. P values were calculated via median regression model. Correlations between expressions of genes/lncRNAs were valued by calculation of Spearman correlation coefficients. Data were analyzed using the R v.4 software and pROC, qreg, and Stan and loo packages. The diagnostic power of the transcript levels of lncRNAs/genes was measured through depicting receiver operating characteristic (ROC) curves.

## Results

### General data of patients with schizophrenia and controls

A total of 50 patients with schizophrenia and 50 healthy subjects were recruited for the current case–control study. Table 1 summarizes the demographic data of cases and controls.

**Table 1:** Demographic data of patients with schizophrenia and controls.

Study groups	Parameters	Values
Patients	Sex (number)	
	Male	33
	Female	17
	Age (Years, mean ± SD (range))	
	Male	51.25 ± 10.38 (32–79)
	Female	46.61 ± 7.37 (31–61)
	Age at onset (Years, mean ± SD (range))	
	Male	34.94 ± 1.86 (29–39)
	Female	35.09 ± 2.47 (29–40)
	Duration (Years, mean ± SD (range))	
	Male	16.73 ± 9.55 (1–46)
	Female	11.52 ± 6.08 (1–22)
	Education (%)	
	Preschool	30%
	School	48.3%
	University	21.7%
Controls	Sex (number)	
	Male	33
	Female	17
	Age (Years, mean ± SD (range))	
	Male	50 ± 12.75 (25–77)
	Female	49.63 ± 8.58 (34–61)
	Education (%)	
	Preschool	11.6%
	School	26.7%
	University	61.7%

### Expression assays

**Figure 1:**
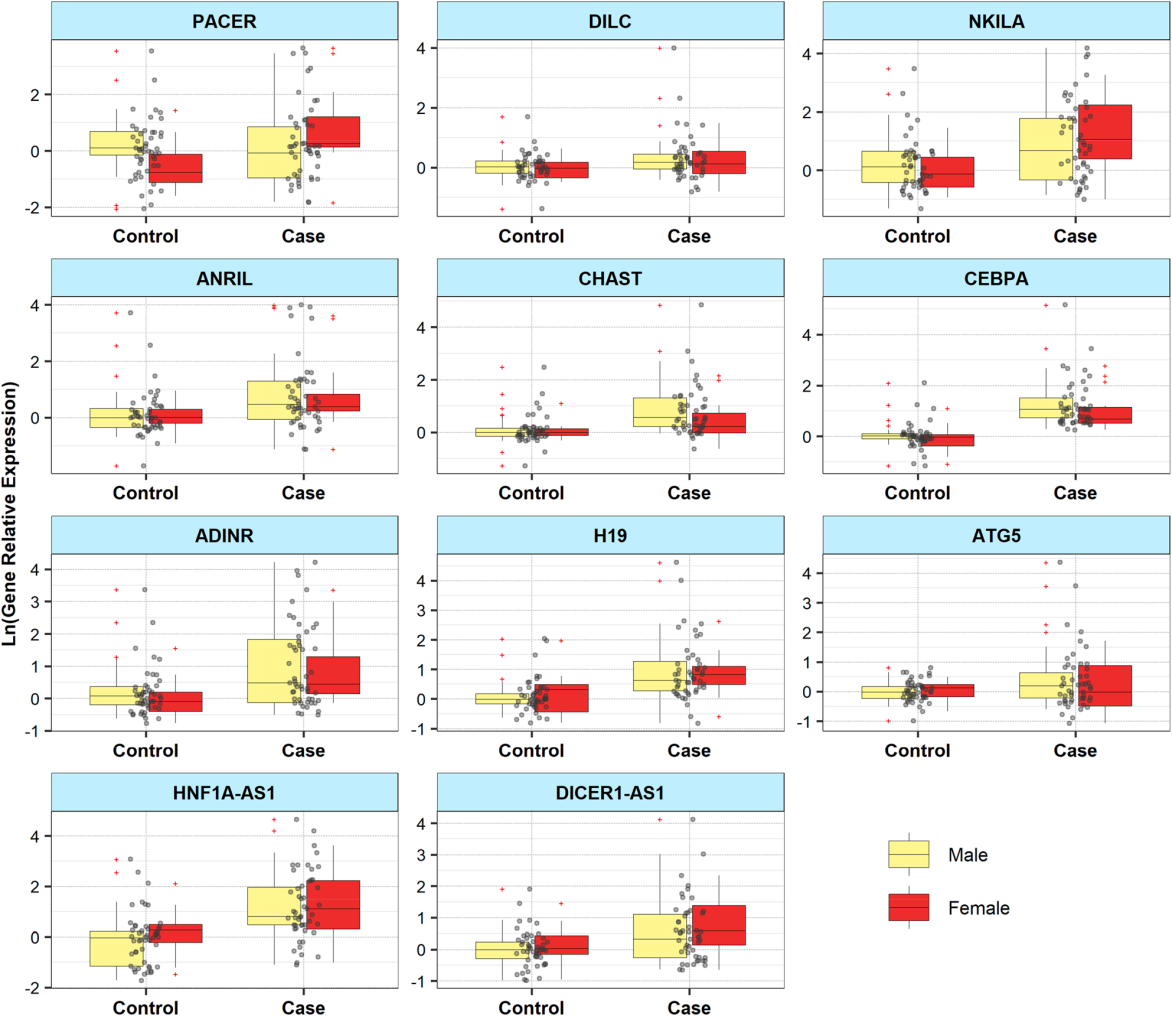
Relative expressions of mRNA coding genes and lncRNAs in patients with schizophrenia and healthy controls. Expression levels of genes were calculated using the Ln (Efficiency^∆Ct) method and are shown as black dots. Mean values and interquartile range are demonstrated. Outliers are shown by+.

Expression levels of mentioned lncRNAs and mRNA coding genes were compared between cases and controls. As demonstrated in Fig. 1, there were significant differences in the expression levels of *PACER*, *CHAST*, *CEBPA*, *H19*, *HNF1A-AS1* and *DICER1-AS1* between cases and controls.

Expression of *PACER* was lower in patients with schizophrenia compared with controls (Posterior beta = − 0.684, P value = 0.049). Group-gender interaction was significant for this lncRNA (P value = 0.005). On the other hand, expressions of *CHAST*, *CEBPA*, *H19*, *HNF1A-AS1* and *DICER1-AS1* were higher in patients compared with controls (Posterior beta = 0.39, P value = 0.005; Posterior beta = 0.844, P value < 0.0001; Posterior beta = 0.467, P value < 0.0001; Posterior beta = 1.107, P value = 0.005; Posterior beta = 0.176, P value = 0.044, respectively). Table 2 shows the results of Bayesian Regression model for comparison of expression of lncRNAs/genes between cases and controls.

**Table 2:** The results of Bayesian Regression model for comparison of expression of lncRNAs/genes between patients with schizophrenia and controls with adjusting the effects of age and gender.

Gene names	Variables	Posterior beta	SE	P value	95% CrI
*DILC*	Group	0.126	0.1	0.188	[− 0.07, 0.33]
	Gender	− 0.108	0.14	0.79	[− 0.36, 0.17]
	Group*Gender	0.045	0.23	0.941	[− 0.44, 0.47]
*NKILA*	Group	0.148	0.18	0.257	[− 0.2, 0.49]
	Gender	− 0.142	0.2	0.567	[− 0.56, 0.25]
	Group*Gender	0.677	0.39	0.461	[− 0.08, 1.45]
*ANRIL*	Group	0.339	0.17	0.09	[− 0.003, 0.67]
	Gender	0.13	0.18	0.87	[− 0.26, 0.44]
	Group*Gender	0.049	0.28	0.736	[− 0.53, 0.61]
*CHAST*	Group	0.39	0.1	0.005	[0.2, 0.6]
	Gender	0.035	0.1	0.974	[− 0.15, 0.22]
	Group*Gender	− 0.32	0.16	0.068	[− 0.65, − 0.01]
*CEBPA*	Group	0.844	0.11	< 0.0001	[0.65, 1.08]
	Gender	− 0.282	0.15	0.762	[− 0.6, 0.003]
	Group*Gender	0.054	0.21	0.116	[− 0.36, 0.47]
*ADINR*	Group	0.109	0.12	0.374	[− 0.13, 0.35]
	Gender	− 0.14	0.15	0.259	[− 0.45, 0.13]
	Group*Gender	0.418	0.2	0.818	[− 0.01, 0.82]
*H19*	Group	0.467	0.12	< 0.0001	[0.26, 0.71]
	Gender	− 0.155	0.28	0.143	[− 0.61, 0.4]
	Group*Gender	0.314	0.33	0.667	[− 0.36, 0.9]
*ATG5*	Group	0.024	0.1	0.141	[− 0.16, 0.22]
	Gender	0.031	0.13	0.367	[− 0.25, 0.28]
	Group*Gender	− 0.393	0.21	0.354	[− 0.84, 0.02]
*HNF1A-AS1*	Group	1.107	0.32	0.011	[0.54, 1.74]
	Gender	0.443	0.36	0.346	[− 0.22, 1.16]
	Group*Gender	− 0.287	0.59	0.999	[− 1.49, 0.87]
*DICER1-AS1*	Group	0.176	0.18	0.044	[− 0.18, 0.55]
	Gender	0.079	0.19	0.898	[− 0.28, 0.45]
	Group*Gender	0.262	0.3	0.531	[− 0.34, 0.84]
*PACER*	Group	− 0.684	0.23	0.049	[− 1.06, − 0.12]
	Gender	− 0.987	0.24	0.006	[− 1.45, − 0.51]
	Group*Gender	1.879	0.36	0.005	[1.12, 2.54]

*SE* standard error, *CrI* Credible Interval, *ER* expression ratio, P values were estimated from Frequentist method.

**Figure 2:**
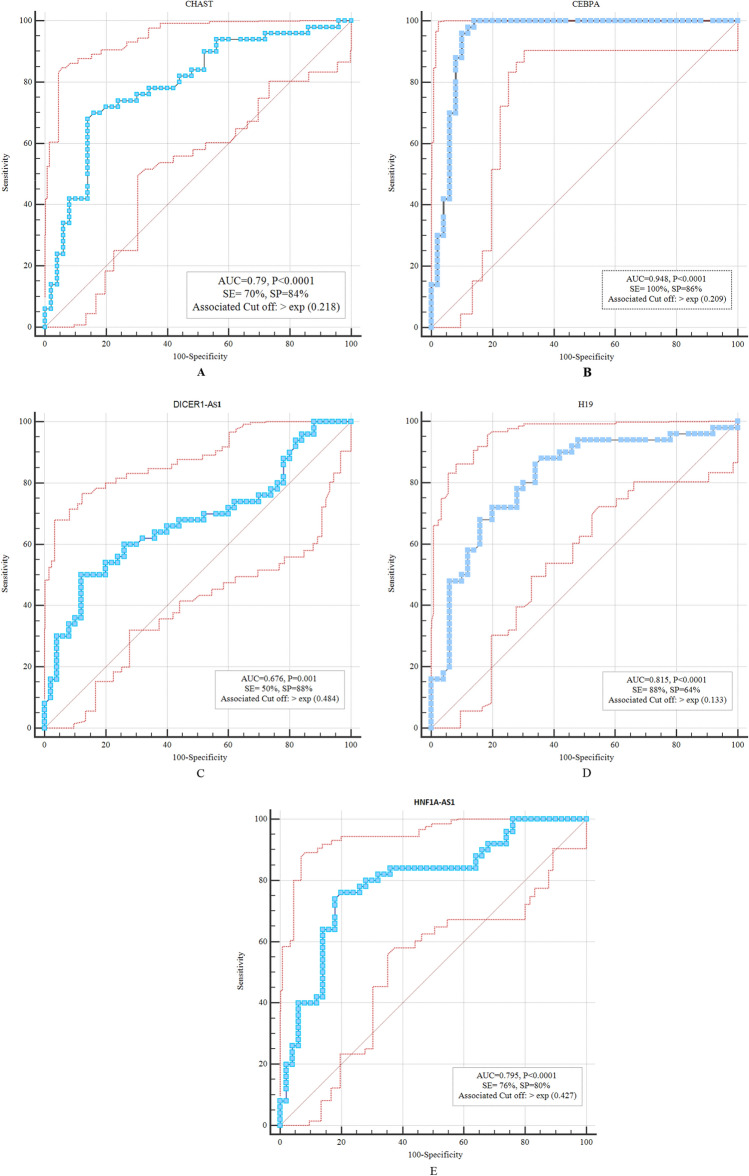
ROC curves showing the diagnostic power of *CHAST* (**A**), *CEBPA* (**B**), *DICER1-AS1* (**C**), *H19* (**D**) and *HNF1A-AS1* (**E**), respectively.

Next, we appraised the diagnostic power of transcript quantities of *CHAST*, *CEBPA*, *DICER1-AS1*, *H19* and *HNF1A-AS1* in distinguishing between patients with schizophrenia and controls through depicting ROC curves (Fig. 2). Based on the area under curve (AUC) values, *CEBPA* had the best diagnostic power (AUC = 0.948, P < 0.0001), followed by *H19* (AUC = 0.815, P < 0.0001).

**Figure 3:**
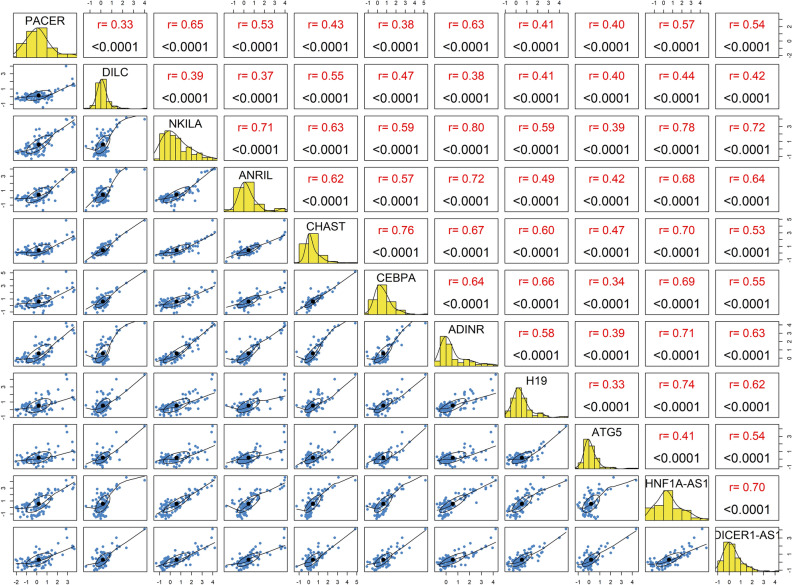
Correlations between expression levels of lncRNAs/genes. The distributions of parameters are depicted on the diagonals. The bivariate scatter plots with a fitted line are displayed on the lower sections of the diagonals. Correlation coefficients and p values of the correlations are shown on the upper parts of the diagonal.

Assessment of pairwise correlation between lncRNAs/genes revealed significant correlation between all pairs. The most robust correlations were observed between *NKILA*/*ADINR* and between *NKILA*/*HNF1A-AS1* (Corerlation coefficients = 0.80 and 0.78, respectively). Figure 3 shows the correlation coefficients and P values.

## Discussion

**Figure 4:**
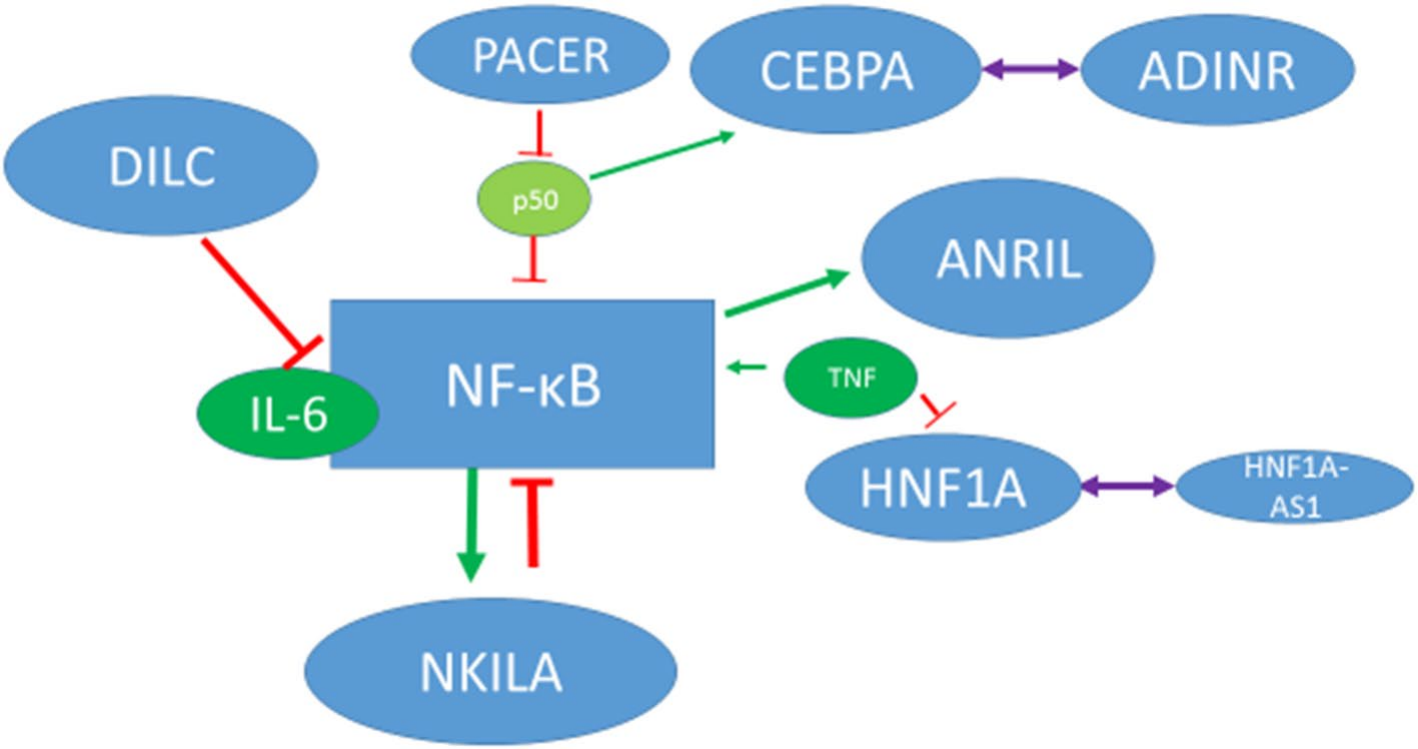
A summary of interaction network between lncRNAs and NF-κB. Inhibitory and activating effects have been shown by red and green arrows, respectively. The interaction between genes and their antisense transcripts are shown in purple.

NF-κB pathway has crucial roles in the pathophysiology of schizophrenia. A recent study in schizophrenia patients has reported up-regulation of the majority of NF-κB family members, the entire NF-κB activation receptors, numerous kinases and IκBα in patients with schizophrenia^27^. This aberrant activity of NF-κB-associated factors has been suggested to be associated with higher levels of cortical immune activation in these patients^27^. Meanwhile, activity and expression of NF-κB-associated genes have been shown to be regulated by lncRNAs^28^. The interaction between lncRNAs and NF-κB-related genes have implications in the pathogenesis of human disorders^28^. In the current study, we evaluated expression of nine lncRNAs and two mRNAs in the peripheral blood of schizophrenic patients and healthy subjects. These genes were previously reported to be linked with NF-κB pathway. Figure 4 depicts a summary of identified interactions between these lncRNAs and NF-κB.

We reported significant down-regulation of *PACER* in patients with schizophrenia compared with healthy subjects. Expression of this lncRNA has been shown to be induced by CTCF^10^. In turn, *PACER* increases expression of COX-2 through blocking suppressive NF-κB complexes^10^. CTCF is possibly involved in the pathogenesis of schizophrenia, since a number of genomic variants within *CTCF* coding genes have been associated with schizophrenia^29^. Besides, assessment of data provided from genome-wide association studies has revealed functional links between schizophrenia risk variants and CTCF binding^30^. Moreover, CTCF has been shown to regulate a primary fate decision step in the construction of cortical interneurons, thus participating in the development of a wide range of neurological conditions including schizophrenia^31^. On the other hand, COX-2 has interactions with a number of neurotransmitters and participates in the modulation of immune responses in the central nervous (CNS) through regulation of production of prostaglandins^32^. Therefore, COX-2 inhibitors have been suggested as therapeutic modalities for some neuropsychiatric conditions such as schizophrenia^32^. The observed down-regulation of *PACER* in patients with schizophrenia in the current study might be due to a possible negative feedback of COX-2 or decreased levels of CFCT. Concomitant assessments of CFTC, *PACER* and COX-2 levels in the peripheral blood as well as central tissues of patients with schizophrenia and functional studies are needed to elaborate the molecular mechanism of involvement of *PACER* in the pathophysiology of schizophrenia. An important finding about this lncRNA was the observed group × gender interaction. While the overall effect suggests that schizophrenia cases have reduced expression of *PACER* relative to controls, this effect appears to be inverted in females. It is possible that the direction of the overall group effect was driven by the higher proportion of males in each group. Therefore, we recommend conduction of studies in larger cohorts of both male and female cases to appraise the group x gender interaction.

On the other hand, expressions of *CHAST*, *CEBPA*, *H19*, *HNF1A-AS1* and *DICER1-AS1* were higher in patients compared with controls. Expression of *CHAST* has been shown to be activated by nuclear factor of activated T cells (NFAT) signaling^11^. The calcineurin-NFAT pathway has acknowledged roles in the normal function of CNS and in the pathobiology of neurological disorders^33^. Particularly, genomic variants within the genes coding for calcineurin isoenzymes have been linked with the susceptibility to schizophrenia^34^. CEBPA interacts with the promoter of leptin coding gene. Receptors for leptin have been detected in several areas of the brain including the hippocampus and cerebral cortex, and are involved in the development of brain and neuroendocrine functions^35^. The role of leptin in the pathobiology of schizophrenia is obscure. While serum levels of this factor are inversely correlated with the severity of positive symptoms in schizophrenia, no correlation has been detected between its concentrations and negative symptoms or cognition^36^. On the other hand, a number of studies have reported correlation between serum leptin levels and better general psychopathology^37,38^. Taken together, the CEBPA-induced alterations in leptin levels have potential roles in at least some aspects of pathobiology of schizophrenia. Alterations in CEBPA levels might also be associated with expression of *H19*. A previous study has shown correlation between DNA methylation in a genomic region near to an important CTCF-binding site in the imprinting control region (ICR) upstream of *H19* and cerebellum weight. Based on these results, authors have suggested an epigenetic mechanism for the development of schizophrenia^39^. *H19* is possibly involved in the pathogenesis of psychiatric disorders through enhancement of neuron apoptosis. An animal study has shown that *H19* increases hippocampal neuronal apoptosis through Wnt signaling^40^. On the other hand, down-regulation of H19 and the *H19*-originated miRNA miR-675 has been associated with over-expression of insulin-like growth factor receptor type 1 throughout the course of neural-like differentiation of stem cells^41^, indicating a possible role for this lncRNA in the neurodevelopment. NF-κB signaling has been shown to inhibit expression of HNF-1α^18^, the transcription factor that is locally related with this lncRNA. Yet, the functional correlation between this transcription factor and *HNF1A-AS1* has not validated^17^. Interestingly, *HNF1A-AS1* has been shown to activate expression of *H19*^17^. Therefore, a possible mechanism for participation of *HNF1A-AS1* in the pathobiology of schizophrenia is its role in induction of *H19* expression. Finally, *DICER1-AS1* has been shown to regulate autophagy via modulation of miR-30b/ATG5 axis^15^. Autophagy has been shown to participate in the physiology of CNS through modulation of neuronal homeostasis. Failure in this process has been associated with the neurologic dysfunction, neurodegenerative disorders and schizophrenia^42^. On the other hand, antipsychotic drugs might ameliorate the observed downregulation of autophagy genes in some parts of brain areas in the schizophrenia patients^42,43^. Therefore, the observed up-regulation of *DICER1-AS1* in patients with schizophrenia might be a compensatory mechanism for enhancement of autophagy in these patients.

We also appraised the diagnostic power of transcript quantities of *CHAST*, *CEBPA*, *DICER1-AS1*, *H19* and *HNF1A-AS1* in distinguishing between patients with schizophrenia and controls through depicting ROC curves. Such aanlyses implied possible use of *CEBPA* and *H19* for this purpose. However, as patients were under treatment with antipsychotic medication, we cannot definitly propose these transcripts as biomarkers. The findings of the present study could help guide future researches, and replication in a sample of drug naive individuals with first-episode psychosis. Such studies would propose possible candidates as biomarkers in schizophrenia.

Finally, assessment of pairwise correlation between lncRNAs/genes revealed significant correlation between all pairs which further supports their participation in a certain signaling pathway namely NF-κB signaling pathway. The most robust correlations were observed between *NKILA*/ *ADINR* and between *NKILA*/ *HNF1A-AS1* which suggest the presence of important functional links between these genes that warrants additional functional analyses.

In brief, the current study demonstrated dysregulation of NF-κB-related lncRNAs and genes in the peripheral blood of patients with schizophrenia and their potential as peripheral markers for this psychiatric condition.

## Supplementary information

Supplementary Information
