# Supplementary material for: Expression of NF-κB associated lncRNAs in schizophrenia
Source: Sci Rep. 2020 Oct 22;10:18105. doi: 10.1038/s41598-020-75333-w (PMC7581809; doi:10.1038/s41598-020-75333-w)
Supplement: Supplementary file 1 — Supplementary Information [file 41598_2020_75333_MOESM1_ESM.docx]

**Expression of NF-κB associated lncRNAs in schizophrenia**

**Amin Safa^1,2^, Elham Badrlou^3^, Shahram Arsang-Jang^4^, Arezou Sayad^3^, Mohammad Taheri^5*^, Soudeh Ghafouri-Fard^3*^**

1. **Institute of Research and Development, Duy Tan University, Da Nang 550000, Viet Nam**

**2. Department of Immunology, School of Medicine, Zabol University of Medical Sciences, Zabol, Iran.**

**3. Department of Medical Genetics, Shahid Beheshti University of Medical Sciences, Tehran, Iran.**

**4. Department of Biostatistics and Epidemiology, Cancer Gene Therapy Research Center, Faculty of Medicine, Zanjan University of Medical Sciences, Zanjan, Iran.**

**5. Urogenital Stem Cell Research Center, Shahid Beheshti University of Medical Sciences, Tehran, Iran.**

**Corresponding authors Mohammad Taheri and Soudeh Ghafouri-Fard**

**Emails: Mohammad_823@yahoo.com and** [**s.ghafourifard@sbmu.ac.ir**](mailto:s.ghafourifard@sbmu.ac.ir)

**Tel & Fax: 00982123872572**

Table S1. Nucleotide sequences of primers used in the current study.

| Gene names | Primer names | Nucleotide sequences | Product size (bp) |
| --- | --- | --- | --- |
| *HNF1A-AS1* | F | CCAGCCTGACCTCTCCATTCC | 158 |
|  | R | GCCGAACTGACATCACTGAACAC |  |
| *NKILA* | F | AACCACTATCATTTTATTTTCCATT | 100 |
|  | R | CAAAGCAATTCTCCTTTCCTA |  |
| *ADINR* | F | TGGATGTGCTGTGATGAAGAGAAG | 91 |
|  | R | CCATAACACCTCCGCAGACAAATC |  |
| *CEBPA* | F | ACTTGGTGCGTCTAAGATGAGG | 144 |
|  | R | CATTGGAGCGGTGAGTTTGC |  |
| *ATG5* | F | TTCGAGATGTGTGGTTTGGAC | 134 |
|  | R | CACTTTGTCAGTTACCAACGTCA |  |
| *CHAST* | F | GCAGAGGGTGCCAACTTGTA | 109 |
|  | R | TCTCAGGGAAATCAGATTGCGG |  |
| *DICER1-AS1* | F | CCCAGCCTGCTTCCTGTTTTAAC | 126 |
|  | R | TTCTCTCCCATCTTCACCTTCTCC |  |
| *DILC* | F | GGAAAGGAGAGAAGAATGG | 144 |
|  | R | GTAAGATGTGGTTGTCGG |  |
| *PACER* | F | TGGTCCTAAGCAGTTACCCTGTA | 177 |
|  | R | ACCAAAATAATCCACGCATCAGG |  |
| *B2M* | F | AGATGAGTATGCCTGCCGTG | 104 |
|  | R | CGGCATCTTCAAACCTCCA |  |
